# Excitability properties of motor axons in adults with cerebral palsy

**DOI:** 10.3389/fnhum.2015.00329

**Published:** 2015-06-03

**Authors:** Cliff S. Klein, Ping Zhou, Christina Marciniak

**Affiliations:** ^1^Guangdong Provincial Work Injury Rehabilitation Center, GuangzhouChina; ^2^Sensory Motor Performance Program, Rehabilitation Institute of ChicagoChicago, IL, USA; ^3^Department of Physical Medicine and Rehabilitation, Feinberg School of Medicine, Northwestern University, Chicago, ILUSA; ^4^Department of Physical Medicine and Rehabilitation, University of Texas Health Science Center at Houston and TIRR Memorial Hermann Research Center, Houston, TXUSA; ^5^Biomedical Engineering Program, University of Science and Technology of China, HefeiChina

**Keywords:** axon, motoneuron, nerve, excitability, myelin, paralysis, cerebral palsy

## Abstract

Cerebral palsy (CP) is a permanent disorder caused by a lesion to the developing brain that significantly impairs motor function. The neurophysiological mechanisms underlying motor impairment are not well understood. Specifically, few have addressed whether motoneuron or peripheral axon properties are altered in CP, even though disruption of descending inputs to the spinal cord may cause them to change. In the present study, we have compared nerve excitability properties in seven adults with CP and fourteen healthy controls using threshold tracking techniques by stimulating the median nerve at the wrist and recording the compound muscle action potential over the abductor pollicis brevis. The excitability properties in the CP subjects were found to be abnormal. Early and late depolarizing and hyperpolarizing threshold electrotonus was significantly larger (i.e., fanning out), and resting current–threshold (I/V) slope was smaller, in CP compared to control. In addition resting threshold and rheobase tended to be larger in CP. According to a modeling analysis of the data, an increase in leakage current under or through the myelin sheath, i.e., the Barrett–Barrett conductance, combined with a slight hyperpolarization of the resting membrane potential, best explained the group differences in excitability properties. There was a trend for those with greater impairment in gross motor function to have more abnormal axon properties. The findings indicate plasticity of motor axon properties far removed from the site of the lesion. We suspect that this plasticity is caused by disruption of descending inputs to the motoneurons at an early age around the time of their injury.

## Introduction

Cerebral palsy (CP) describes a group of permanent disorders of movement and posture causing activity limitations that are attributed to non-progressive disturbances that occur in the developing fetal or infant brain (Rosenbaum et al., 2007). Consistent with an upper motoneuron syndrome, the predominant consequence of CP is motor impairment. People with CP present with a mix of clinical features of different severity including difficulties in movement, balance, and coordination associated with reduced voluntary strength and spasticity (Koman et al., 2004). Although the lesion itself is non-progressive, the clinical features evolve with increased age. Indeed, there is evidence that motor function may deteriorate during adulthood (Sandstrom et al., 2004). Given that the number of adults with CP is rising, there is a need for more specialized care and research in this population, including studies that can address the neurophysiology mechanisms underlying motor impairment (Peterson et al., 2012).

Conditions such as CP and stroke alter the amount of descending excitatory and inhibitory inputs to the spinal cord (Eyre et al., 2000; Rose and McGill, 2005; Klein et al., 2010). For example, a loss of excitatory corticospinal input likely reduces motoneuron recruitment and firing rate (Eyre et al., 2000; Rose and McGill, 2005; Hussain et al., 2014). Simultaneously, a reduction in inhibitory inputs would tend to heighten motoneuron excitability (Barolat-Romana and Davis, 1980; O’Sullivan et al., 1998; Achache et al., 2010). Although the brain injury is the root cause of motor impairment in CP, disrupted descending inputs (i.e., decentralization) may lead to secondary changes in the spinal cord and periphery (i.e., motoneurons, axons, and muscle fibers) causing further deterioration in function (Eyre et al., 2000; Peterson et al., 2012; Marciniak et al., 2015). Intrinsic motoneuron and peripheral axon properties are altered in stroke (Jankelowitz et al., 2007a; Suresh et al., 2014), but whether this is the case in CP is uncertain. In one study, lower limb nerve conduction velocity in children with CP and healthy adults was compared and found not to be different (Takebe and Basmajian, 1976). However, another group reported slower sensory and motor conduction velocities in the more affected than the less affected limb in children with CP, but comparisons to healthy controls was not done (Lang et al., 1983). Given that one goal of neurorehabilitation in people with central motor disorders is to establish better control of peripheral motor networks, it is crucial to understand how the peripheral machinery has been altered as a result of decentralization and disuse (Dobkin, 2004).

Measures of axon excitability may provide important clues into the molecular processes underlying motoneuron (axonal) plasticity associated with disease, injury, and disuse (Bucher and Goaillard, 2011). This notion is reasonable given that structures crucial to axonal function are derived in part from synthetic activities of the motoneuron (Weiss and Hiscoe, 1948). Axon excitability is largely determined by the combined effects of active (i.e., ion channels and pumps) and passive (i.e., axonal size, myelin) properties. Insight into the biophysical properties of peripheral axons *in vivo* can be obtained with nerve excitability testing using threshold tracking (Bostock et al., 1998). With this technique, investigators have reported significant changes in membrane potential, ion conductances, and passive properties of peripheral axons in a variety of conditions including central motor disorders (Boerio et al., 2007; Jankelowitz et al., 2007a; Lin et al., 2007; Ng et al., 2008; Krishnan et al., 2009).

In the present study, we examined whether CP causes changes in the excitability properties of motor axons. Nerve excitability testing was completed in a group of adults with CP and a group of healthy controls. A variety of nerve excitability tests were conducted to indirectly assess nodal and internodal properties, including ion conductance and the resting membrane potential. Given the evidence that motoneurons are hyperexcitable in CP, we anticipated that motor axons would also be hyperexcitable, and more so in those with greater motor impairment.

## Materials and Methods

Seven adults with CP ranging in age from 20 to 45 years (mean ± SE, 32.3 ± 3.0 years) were studied. Five were quadriplegic, one was triplegic, and one was diplegic (**Table 1**). They were recruited from outpatient clinics at the Rehabilitation Institute of Chicago and the Cerebral Palsy Research registry of the Department of Physical Therapy and Human Movement Sciences at Northwestern University (Chicago, IL, USA). A control group of 10 men and 4 women 40–59 years (50.5 ± 1.6 years) was also studied. The study was approved by the Institutional Review board of Northwestern University and all subjects gave their written consent prior to the experiments.

**Table 1:** Demographic and clinical information of the cerebral palsy (CP) subjects.

ID	G	Age	Test side	P	GMFCS	MACS	MAS EF	MAS WF	MAS FF	MRC EF	MRC FF	MRC APB	HG MVC(%)	KP MVC(%)
(1)	F	27	R	Q	III	I	2	2	2	4+	4	4−	31.9	37.5
(2)	F	33	L	Q	III	II	2	1	0	5	5	4−	84.4	90.1
(3)	F	45	L	Q	IV	III	–	–	–	5	5	3	27.6	41.3
(4)	F	20	R	D	II	II	0	1+	0	5	5	4+	88.1	112.5
(5)	M	40	R	T	I	I	1	0	0	5	5	5	84.5	77.5
(6)	F	28	L	Q	IV	II	0	0	0	5	5	4	116.3	106.6
(7)	M	27	L	Q	I	II	1	0	0	5	4	3	27.9	44.2

*ID, subject ID number; G, gender; F, female; M, male; R, right; L, left; P, pattern of injury; Q, Quadriplegia; T, Triplegia; D, Diplegia; GMFCS, Gross Motor Function Classification System; MACS, Manual Ability Classification System; MAS, Modified Ashworth Scale; MRC, Medical Research Council manual muscle strength; EF, elbow flexors, WF, wrist flexors, FF, finger flexors; APB, abductor pollicis brevis; HG, hand grip; KP, key pinch; MVC(%), Maximal voluntary contraction force as a percentage of age and sex matched norms (Mathiowetz et al., 1985). Note some measurements were not recorded in subject number 3.*

### Clinical Measurements

A physician (CM) completed a clinical examination of each CP subject. Motor function was determined using the Gross Motor Function Classification System (GMFCS; Palisano et al., 1997). In the GMFCS, motor function is divided into five levels, with level I (‘Walks without limitations’) reflecting the highest level of function and level V (‘Transported in a Manual Wheelchair’) the lowest. Although the GMFCS was developed for use in children, it has been applied in adults with CP, and was found to correlate with activities of daily living (i.e., functional independence measure; Sandstrom et al., 2004; Liu et al., 2014). The ability to handle objects was assessed using the Manual Ability Classification System (MACS; Eliasson et al., 2006). The MACS classifies the collaborative use of both hands working together into five levels, with level I (‘Handles objects easily and successfully’) the highest level of function and level V (‘Does not handle objects and has severely limited ability to perform simple actions’) the lowest. Spasticity and motor power of the upper limb were assessed using the Modified Ashworth Scale (MAS; Bohannon and Smith, 1987) and Medical Research Council (MRC) scales, respectively.

### Maximal Voluntary Contraction (MVC)

Two measures of maximal voluntary contraction (MVC) strength were recorded using dynamometry in the same arm used for excitability testing. One was the force produced during hand grip (Jamar Plus hand dynamometer, Sammons Preston, Bolingbrook, IL, USA) and the other was the force produced during key pinch (pinch gage, model PG-60, B&L Engineering, Santa Ana, CA, USA; Mathiowetz et al., 1985). Three MVCs were recorded for each maneuver, each lasting about 4 s, with at least 1 min rest between contractions, and the largest value is reported in the results. The order in which grip and pinch MVC was recorded was balanced across subjects.

### Nerve Excitability Testing

Nerve excitability testing was completed on one arm of the CP participants and the right arm of the controls. The more affected side in the CP subjects was chosen based on their perception of which arm most limited their daily activities. However, there was little difference between the right and left arms according to the clinical examination. The median nerve was stimulated via surface electrodes, with the cathode near the proximal wrist crease and the anode 10 cm proximally. The pulse duration of all stimuli was 1 ms, except when recording strength-duration properties. The evoked compound muscle action potential (CMAP) was recorded over the abductor pollicis brevis muscle, with the active electrode over the motor point and the reference over the metacarpal phalangeal joint. Non-polarizable disposable silver–silver chloride electrodes (NeuroPlus 2.5 cm × 2.5 cm, Vermed, Bellows Falls, VT, USA) were used for stimulation and EMG recording. A ground electrode (1 cm metal disk) was placed on the dorsal surface of the hand. The raw EMG was amplified (×500), bandpass filtered (10 Hz–3 kHz; Astro-Medical, model P511, West Warwick, RI, USA), and digitized at a sampling rate of 10 kHz with a 16-bit data acquisition system (NI-USB6221; National Instruments; Austin, TX, USA). Stimulation and recording were controlled by QTracS software (©Professor H. Bostock, Institute of Neurology, London). The stimulus waveforms generated by the computer were converted to current via a constant current linear bipolar stimulator (DS5, Digitimer Ltd., Welwyn Garden City, Hertfordshire, UK). Skin temperature was monitored (Omega Thermistor Thermometer, Omega Engineering Inc., Stamford, CT, USA) close to the cathode, and was kept at temperatures of 32°C or above by covering the arm with towels.

The basis of the axon excitability technique is the tracking of threshold current required to evoke a predetermined CMAP amplitude (i.e., ‘threshold tracking’). In the present study, the threshold current needed to evoke a 40% CMAP (i.e., the test or control pulse) was tracked before, during, and after different maneuvers designed to assess nodal and internodal properties, i.e., the Trond protocol (Kiernan et al., 2000). These maneuvers consisted of changing the pulse duration to assess the strength-duration properties, applying long-duration subthreshold polarizing conditioning currents to determine threshold electrotonus and the current–voltage relationship, and applying supramaximal stimuli to assess the recovery cycle. Changes in threshold current resulting from these maneuvers were expressed as a percentage of the threshold current of the test pulse. The five components of the Trond protocol were recorded in the following order:

#### Stimulus–Response Curve

A stimulus–response curve is the graphic display of CMAP amplitude versus stimulus intensity; from minimal current up to current levels required to evoke the maximum CMAP. These responses were recorded in order to set the target 40% CMAP to be used during threshold tracking. In addition, the slope of the curve, together with the tracking error (deviation from target), was used to optimize the tracking step, i.e., the amount by which the stimulus is automatically increased or decreased if the response is too small or too large, respectively.

#### Strength-Duration Properties

The threshold of the test pulse was recorded intermittently (2 Hz) with threshold to another stimulus whose duration was reduced in 0.2 ms steps from 1 ms down to 0.2 ms. The threshold stimulus charge was plotted against stimulus duration for the five different durations (0.2, 0.4, 0.6, 0.8, and 1.0 ms). The strength-duration time constant (SDTC) is equal to the negative intercept on the *x*-axis, and the rheobase is equal to the slope of the line. The SDTC depends primarily on nodal resistive–capacitive properties as well as persistent Na^+^ currents active at rest (Bostock and Rothwell, 1997).

#### Threshold Electrotonus

Prolonged (100 ms) subthreshold polarizing currents were used to alter the nodal and internodal membrane potential, a process referred to as electrotonus. The term “threshold electrotonus” refers to the fact that changes in threshold usually parallel the underlying electrotonic changes in membrane potential (Bostock and Baker, 1988). Five stimulus combinations were tested in turn; test (control) stimulus alone, test stimulus plus 100 ms polarizing conditioning current set to ±40% of the control threshold current; test stimulus plus 100 ms polarizing conditioning set to ±20%. Each stimulus combination was repeated until three valid threshold estimates were recorded. A valid threshold was defined as the CMAP being within 15% of the target response, or alternate responses being above or below the target (Kiernan et al., 2000).

#### Current–Threshold Relationship

The current–threshold relationship is a threshold analog of the current–voltage relationship (I/V), and quantifies the rectifying properties of the internode. This relationship was determined by recording the threshold for the target CMAP at the end of 200 ms subthreshold polarizing currents of different strengths. The strength of the polarizing current was adjusted in 10% steps from +50% of control threshold (depolarizing) to -100% of control threshold (hyperpolarizing). The criteria for a valid threshold estimate was the same as that used during recording of threshold electrotonus.

#### Recovery Cycle

Following the discharge of an action potential, axons undergo a sequence of changes in excitability referred to as the recovery cycle. The recovery cycle following a supramaximal conditioning stimulus was tested at 18 conditioning-test intervals, decreasing from 200 to 2 ms. The CMAP produced by the supramaximal conditioning stimulus was subtracted online from the response to the conditioning-test pair so that the test CMAP could be measured accurately when the conditioning-test interval was short. Each conditioning-test pair was repeated until four valid threshold estimates were recorded.

### Mathematical Modeling

Interpretation of group differences in nerve excitability was aided by a mathematical model (the ‘Bostock model’) of the human motor axon (Bostock et al., 1991; Kiernan et al., 2005; Kanai et al., 2006; Jankelowitz et al., 2007a; Howells et al., 2012; Tomlinson et al., 2012; Boerio et al., 2014). The Bostock et al. (1991) model is based on a single node and internode connected by an internodal leak pathway (the Barrett–Barrett conductance-GBB). The computer program MEMFIT, which is part of the QtracP data analysis program (©UCL Institute of Neurology), was used to test the effects of changes in different excitability parameters (i.e., ion conductances, GBB) on the goodness-of-fit of the model to the recorded excitability data. The modeling involved iterative changes of single parameters or combinations of parameters to minimize the ‘discrepancy’ between the simulated excitability measures and the recorded excitability measures of the Trond protocol. The parameters tested were the total nodal Na^+^ currents (transient and persistent), K^+^ (slow and fast at node and internode), leak conductances (node and internode), GH, and GBB. At times corresponding to those in the excitability recordings, the excitability of the model nerve was tested repeatedly to determine threshold with an accuracy of 0.5%. The ‘discrepancy’ between the model and the recordings was obtained by weighting the errors of four Trond components as follows: strength-duration data, 0.5; threshold electrotonus, 1; current–threshold, 1; recovery cycle, 1. The reported results are based on optimizing the fit to the mean data as closely as possible as we found that this procedure provided a better fit than weighting each data point according to its SD (Howells et al., 2012). The excitability data for the control group were modeled first using the standard model available in the QtracP software (i.e., NC29 parameters; Kiernan et al., 2000). In this case, the program made small changes of up to three parameters at a time until the fit could not be made any better. The CP excitability data were then fit using the control group parameters as the template. The model was run in unclamped mode so that secondary changes in resting membrane potential could occur in response to changes in conductances or pump currents.

### Data Analysis

The following parameters were determined from each recording for numerical analysis using the QtracP program (Kiernan et al., 2000; **Table 2**). The peak CMAP was the mean of the last three points on the stimulus–response curve. Latency was the time between the stimulus onset and the onset of the largest CMAP. The stimulus–response slope was estimated from the normalized stimulus–response relationship as the stimulus evoking a 75% maximal response minus that evoking a 25% maximal response, divided by that evoking a 50% maximal response. A number of parameters were derived from the threshold electrotonus recording. The TEd(peak)%, TEd(90–100 ms)%, TEd(undershoot)%, were the mean threshold reductions at the specified point, or between the specified latencies during depolarizing conditioning, and the TEh(10–20 ms)%, TEh(90–100 ms)%, TEh(overshoot)%, were the corresponding parameters during hyperpolarizing conditioning. From the current–threshold relationship, the resting I/V slope was calculated from polarizing currents between -10 and +10% of resting threshold. The minimal I/V slope was calculated by fitting a straight line to each three adjacent points in turn. Four parameters of the recovery cycle are presented. Superexcitability (%) was the minimum mean of three adjacent points and subexcitability (%) was the maximum mean after 10 ms. The relative refractory period (RRP) was the first intercept on the *x*-axis, and refractoriness at 2.5 ms was the threshold increase (%) at the 2.5 ms conditioning-test interval.

**Table 2:** Multiple measures of axon excitability in CP and control groups.

Excitability measure	CP	Control	*P*-value
**+20% depolarizing electrotonus**
TEd(peak) (%)	43.2 ± 1.3	38.7 ± 9.5	0.008
TEd(90–100 ms) (%)	31.0 ± 1.0	28.0 ± 0.6	0.01
TEd(undershoot) (%)	−12.2 ± 1.0	−10.0 ± 0.8	0.14
**+40% depolarizing electrotonus**
TEd(peak) (%)	73.7 ± 1.4	69.4 ± 1.2	0.04
TEd(90–100 ms) (%)	49.2 ± 2.2	46.8 ± 0.8	0.2
S2 accommodation (%)	24.0 ± 2.2	22.6 ± 1.2	0.56
TEd(undershoot) (%)	−23.3 ± 1.3	−18.0 ± 1.2	0.01
**−20% hyperpolarizing electrotonus**
TEh(10–20 ms) (%)	−42.4 ± 1.4	−37.6 ± 0.6	0.001
TEh(90–100 ms) (%)	−58.4 ± 3.9	−47.5 ± 1.8	0.009
TEh(overshoot) (%)	10.8 ± 0.5	8.2 ± 0.7	0.02
**−40% hyperpolarizing electrotonus**
TEh(10–20 ms) (%)	−84.7 ± 2.7	−75.8 ± 1.1	0.002
TEh(90–100 ms) (%)	−143.9 ± 8.6	−120.7 ± 4.3	0.01
TEh(overshoot) (%)	15.9 ± 1.0	14.0 ± 1.0	0.3
**I/V relationship**
Depolarizing slope	0.95 ± 0.02	1.0 ± 0.02	0.05
Resting I/V slope	0.52 ± 0.03	0.58 ± 0.01	0.06
Minimum I/V slope	0.22 ± 0.01	0.23 ± 0.01	0.6
Hyperpolarizing I/V slope	0.38 ± 0.02	0.35 ± 0.02	0.36
**Recovery cycle**
RRP (ms)	2.80 ± 0.19	3.04 ± 0.11	0.26
Refractoriness at 2.5 ms (%)	8.6 ± 8.0	23.0 ± 5.5	0.18
Superexcitability (%)	−26.3 ± 1.9	−22.6 ± 1.2	0.12
Subexcitability (%)	13.5 ± 3.0	13.4 ± 1.2	0.93

### Statistics

Mean values in the CP subjects were compared to the mean values in the controls using two-tailed Student’s *t*-tests. Pearson’s product-moment correlation coefficients were applied to determine the relationships between different excitability parameters as well as between clinical measures and excitability parameters. Differences were considered significant when *P* < 0.05, and data are presented as means ± SE.

## Results

### Clinical Features

The clinical measurements and MVC of the arm used for nerve excitability testing are summarized in **Table 1**. The GMFCS was level III or above in four of the seven CP subjects indicating significant impairment in gross motor function. The MACS was level II or above in five subjects reflecting impairment in ability to manipulate objects. About half of the group had mild spasticity of the elbow flexors, wrist flexors, and finger flexors based on the MAS scores. Weakness was more prevalent in the abductor pollicis brevis than the finger flexors or elbow flexors according to the MRC scale. None of the clinical measures (GMFCS, MACS, MRC, MAS) were found to be significantly correlated with one another.

### Maximal Voluntary Contraction

Maximal strength was measured with hand dynamometry to quantify weakness more precisely. The mean pinch MVC was smaller in the CP than the control group (6.3 ± 1.0 and 9.4 ± 0.6 kg, *P* = 0.01) as was the grip MVC (23.5 ± 5.1 and 41.4 ± 3.4 kg, *P* = 0.007). Given the differences in age and the proportion of men and women between the two groups, the MVC of each CP subject was expressed as a percentage of normative values (i.e., matched according to age, sex, and side tested) previously recorded with the same model dynamometers (Mathiowetz et al., 1985). Three CP subjects demonstrated significant pinch and grip weakness (MVC = ∼25–45% of normative values), whereas four others had normal or near normal strength (MVC = ∼85–112%; **Table 1**). The mean pinch and grip MVC forces of the CP subjects were 72.8 ± 12 and 65.8 ± 13.6% of the normative data, respectively. The absolute pinch and grip MVCs (kg) were highly correlated among the seven CP subjects (*R* = 0.92, *P* = 0.004) as were the relative (% of normative data) values (*R* = 0.94, *P* = 0.002). Also, the absolute pinch (*R* = 0.74, *P* = 0.05) and grip (*R* = 0.83, *P* = 0.02) MVCs were correlated with the abductor pollicis brevis MRC scores. The absolute pinch and grip MVCs were also highly correlated among the 14 controls (*R* = 0.87, *P* = 0.00006).

### Nerve Excitability Testing

All components of the Trond protocol were completed in all control subjects. For the most part the CP subjects also tolerated the procedures well. However, one subject (number 3) could not tolerate the stimuli of the current–threshold and recovery cycle recordings, and another (number 6) also could not tolerate the recovery cycle. Hence, the reported mean data derived from the current–threshold relationship and recovery cycle recordings are based on six and five subjects, respectively. The skin temperature at the stimulation site did not differ between CP and control groups; 32.8 ± 0.3° and 33.0 ± 0.2°, respectively.

### Threshold Electrotonus

**FIGURE 1:**
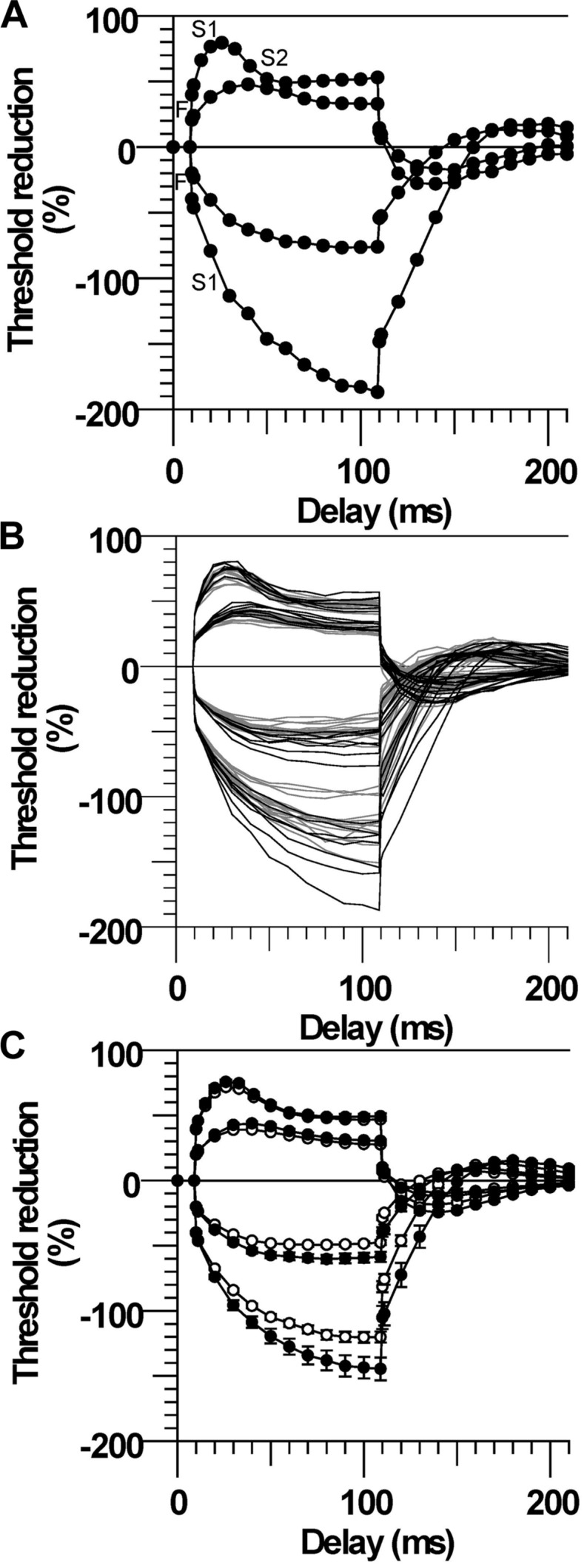
**Larger threshold electrotonus in cerebral palsy (CP). (A)** Threshold changes during 100 ms ±20 and ±40% threshold currents in one CP subject (number 2). The different phases of electrotonus (F, S1, and S2) are noted for the ±40% threshold responses. **(B)** Responses of all CP (black lines) and control (gray lines) subjects. **(C)** Mean (±SE) responses demonstrating the larger changes in early and late threshold electrotonus (“fanning out”) in CP (filled symbols) compared to control subjects (unfilled symbols).

The most conspicuous abnormality recorded in the CP subjects was their larger change in threshold, referred to as ‘fanning out’ due to the resemblance to ribs of a Japanese fan (Nodera et al., 2004), during the ±20 and ±40% subthreshold conditioning currents. The CP subject with the largest fanning out is depicted in **Figure 1A**. By convention, the changes in threshold are plotted as threshold reductions, with the responses to depolarization plotted upward (indicating an increase in excitability) and the responses to hyperpolarization plotted downward (indicating a decrease in excitability). At onset of the depolarizing stimulus there is a rapid reduction in threshold due to nodal depolarization (F phase). This is followed by a slower period of threshold reduction reflecting spread of depolarizing current from the node to the internode (S1 phase). The threshold then starts to return slowly toward the control level indicating accommodation due to activation of slow K^+^ currents (S2 phase). At the onset of the hyperpolarizing stimulus there is also rapid increase in threshold (F), followed by a longer and slower increase (S1).

Threshold electrotonus for all subjects is plotted in **Figure 1B** and the group means in **Figure 1C**. The early and late changes in threshold elicited by hyperpolarizing electrotonus were larger in most CP compared to control subjects; i.e., the threshold increases 90–100 ms after the onset of the -40% current were larger than the control group mean in six of the seven subjects, and the group means were different (TEh90–100 ms, *P* = 0.01, **Table 2**). The responses 90–100 ms after the onset of the -20% current pulse were similarly larger in CP (*P* = 0.009). The reductions in threshold elicited by depolarizing electrotonus were also larger in CP than control; i.e., peak reductions in threshold during 20 and 40% currents were above the control means in six of the seven CP subjects, and the group means were different (TEdpeak, *P* = 0.008 and 0.04, respectively, **Table 2**). The threshold reductions 90–100 ms after the onset of the 40% current pulse were not significantly different between the groups (*P* = 0.2). Note that data for the late 40% current (after 20 ms) for one CP subject was excluded because of irregularity of the threshold responses, possible due to activation of some low threshold axons during the conditioning pulse.

### Current–Threshold Relationship

**FIGURE 2:**
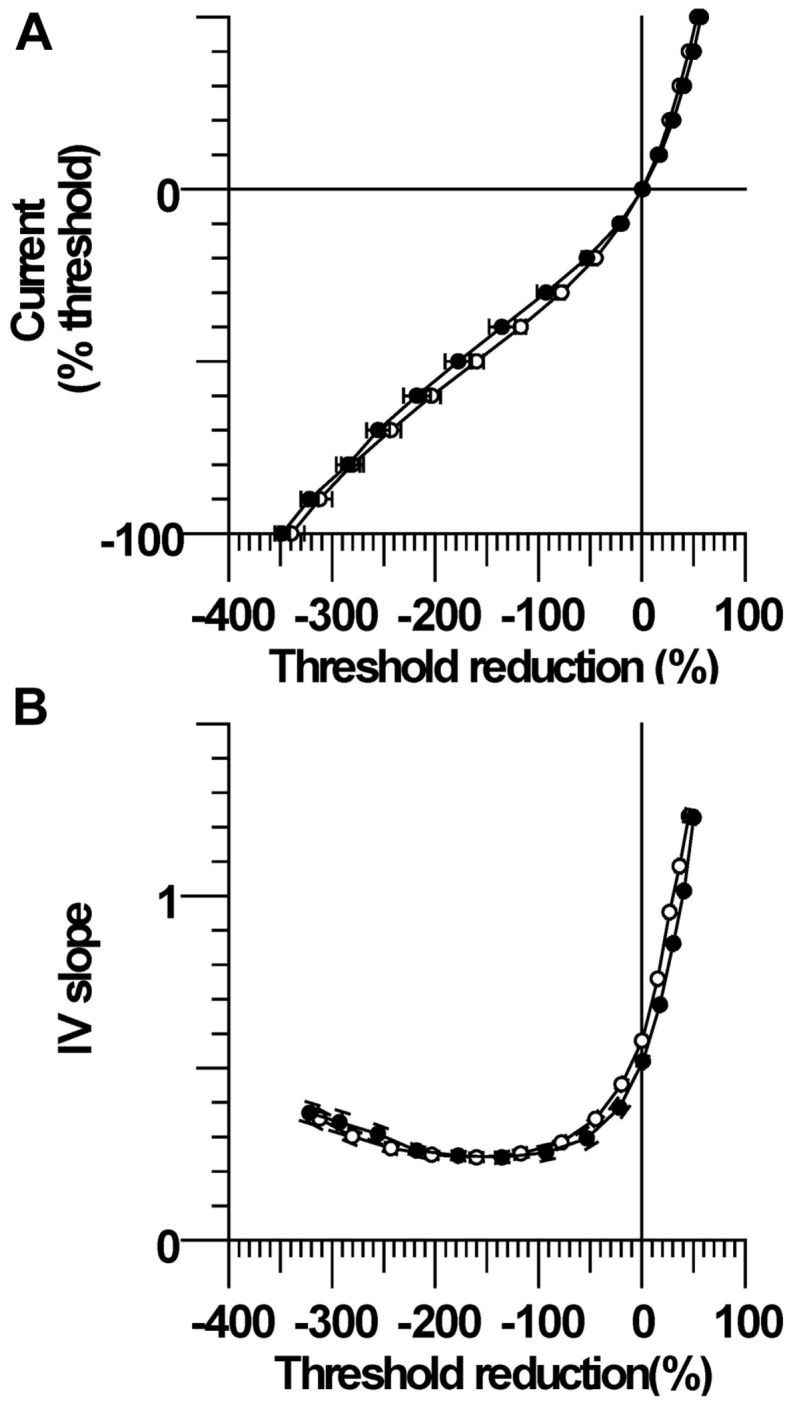
**Current–threshold relationship and IV slope. (A)** Threshold increases were significantly larger in CP than control during modest strength hyperpolarizing currents (-10 and -20%). Threshold reductions were significantly larger in CP than control during 10–40% depolarizing currents. **(B)** The resting I/V slope and the depolarizing I/V slope were smaller in CP than control.

The changes in threshold at the end of the 200 ms polarizing pulse of different current strengths were plotted to form the current–threshold relationship, a threshold analog of the current–voltage (I/V) relationship (**Figure 2A**). Like a conventional I/V plot, the reduction in threshold during membrane depolarization is plotted to the right and the increase in threshold during membrane hyperpolarization is plotted to the left. The steepening curve to the top right reflects increasing outward rectification due to activation of K^+^ channels, whereas steepening of the curve to the bottom left represents increasing inward rectification due to the hyperpolarization-activated cation current (Ih). There were larger threshold changes in the CP subjects during weak depolarizing (+10 to +40% threshold) and hyperpolarizing (-10 to -20%) currents, consistent with the group differences in threshold electrotonus (*P* < 0.05). However, threshold changes during stronger hyperpolarization (-30 to -100%) were not different between CP and control (*P* > 0.1), which likely means that activation of Ih at these current strengths was not different between groups. The calculated slopes of the current–threshold relationship (I/V slope) provide a clearer indication of differences in resting input conductance and rectification (**Figure 2B**). The calculated resting slope (i.e., between -10 and +10% threshold) and depolarizing slope were smaller in the CP than control (*P* = 0.06 and *P* = 0.05, **Table 2**). Neither the minimum slope nor hyperpolarizing slope differed between the groups (**Table 2**).

### Stimulus–Response Relationship

**FIGURE 3:**
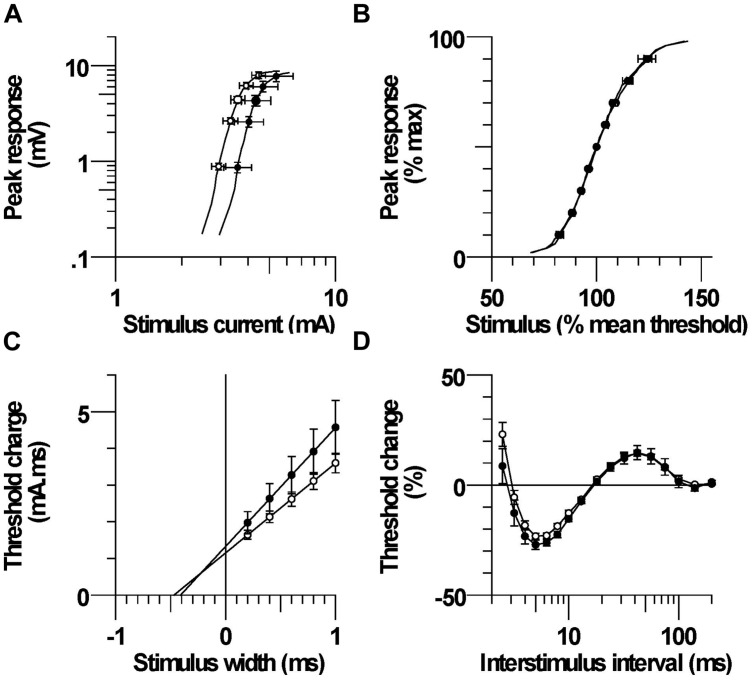
**(A)** Mean stimulus–response relationship in CP and control subjects. **(B)** Normalized stimulus–response relationship. **(C)** Mean threshold charge versus stimulus width. **(D)** Recovery cycle consisting of refractory, superexcitability, and subexcitability periods.

The mean maximum CMAP onset latency was not significantly different between CP and control; 3.74 ± 0.19 ms (3.0–4.5 ms) and 3.61 ± 0.15 ms (2.9–4.9 ms), respectively, (*P* = 0.6). The corresponding maximum CMAP amplitude was also not significantly different between groups; 9.0 ± 1.2 mV (5.3–15.5 mV) and 9.2 ± 0.7 mV (4.8–14.1 mV; *P* = 0.9). Larger absolute currents were generally required to evoke a given absolute CMAP in CP compared to control, but the stimulus required to evoke 50% of the maximum CMAP was not significantly different; 4.65 ± 0.70 mA and 3.72 ± 0.28 mA, respectively (*P* = 0.2, **Figure 3A**). Stimulus–response curves were normalized by expressing the potentials as a percentage of the maximum CMAP and the stimuli as percentages of the current for a response 50% of maximum. The normalized stimulus response slopes were similar between CP and control; 5.10 ± 1.1 and 4.74 ± 1.0, *P* = 0.5, **Figure 3B**).

### Strength-Duration Properties

The mean threshold charge versus stimulus width is shown in **Figure 3C**. The threshold charge was larger in CP than control at each of the five pulse widths (0.2, 0.4, 0.6, 0.8, 1.0 ms), but the differences were not significant (*P* = 0.14–0.23). The rheobase, corresponding to the slope of the line was larger in CP than control, but the difference was not significant; 3.23 ± 0.53 and 2.45 ± 0.19, respectively (*P* = 0.16). The SDTC, corresponding to the negative intercept on the *x*-axis, was also not significantly different between CP and control; 0.423 ± 0.027 ms and 0.478 ± 0.027 ms, (*P* = 0.2).

### Recovery Cycle

After the discharge of an action potential, axons undergo a sequence of excitability changes referred to as the recovery cycle. As a consequence of Na^+^ channel inactivation, threshold is increased immediately after discharge and returns to control values in 3–4 ms (i.e., refractory period). A period of increased excitability (superexcitability) ensues that is primarily determined by the depolarizing after potential (Barrett and Barrett, 1982). Superexcitability is followed by a period of decreased excitability (subexcitability) that reflects activation of slow K^+^ channels. The recorded refractoriness at 2.5 ms and the RRP were both smaller in CP than control, but the differences were not significant (*P* = 0.18 and 0.26, **Figure 3D**; **Table 2**). Superexcitability tended to be larger in CP than control (*P* = 0.12), whereas subexcitability was similar between groups.

### Mathematical Modeling of Axon Excitability

**FIGURE 4:**
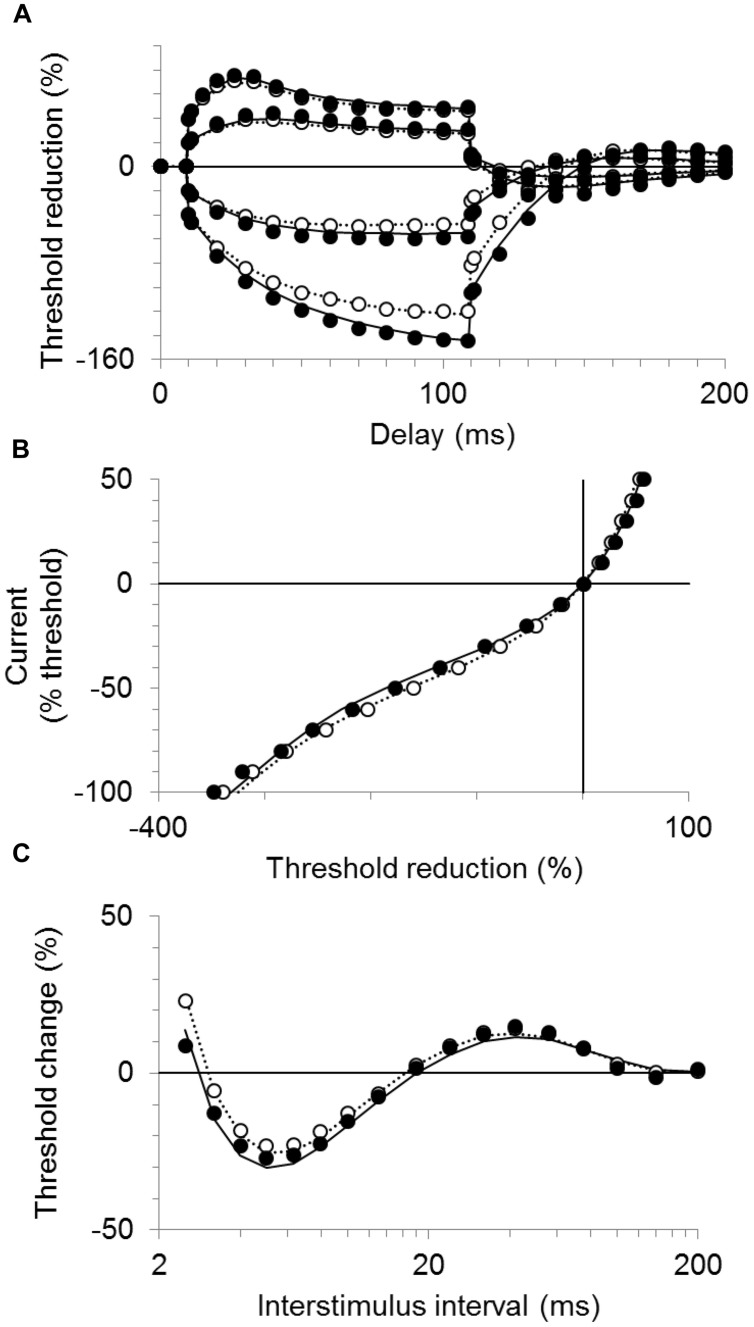
**Modeling results for the CP and control subjects**. The mean responses are replotted for the CP group (filled symbols) and control group (unfilled symbols) together with the best fit simulations (solid and dotted lines, respectively). **(A)** Threshold electrotonus, **(B)** current–threshold relationship, and **(C)** recovery cycle.

**FIGURE 5:**
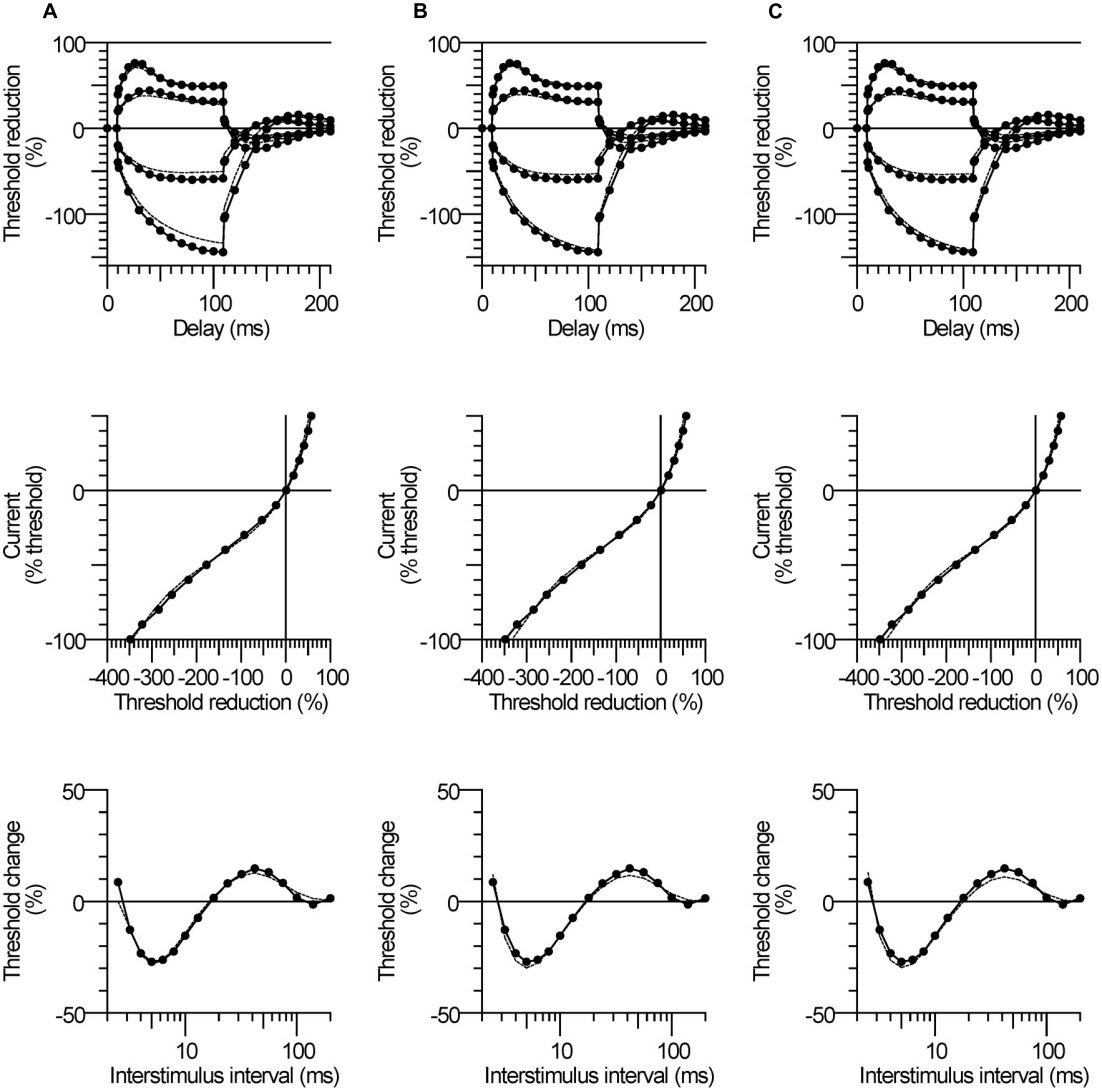
**Modeling results for the CP subjects**. The mean responses of threshold electrotonus, I/V relationship, and recovery cycle (top to bottom) are replotted for the CP group (filled symbols) together with the best fit simulations (dashed lines) corresponding to listed parameters in **Table 3B**; **(A)** an increase in GBB together with a reduction in GH to 5.15, **(B**) an increase in GBB together with an increase in GKsN to 42.5, **(C)** an increase in GBB together with a decrease in PNaN to 4.05.

**Table 3** lists the best fits of the motor axon model to the mean CP excitability data by changing one or two parameters at a time. The best single parameter fit was obtained by increasing GBB by 6% (from the control group value of 35.8 units to 38 units). This resulted in a 62% reduction in discrepancy between the model and the recorded waveforms for the CP subjects. Allowing two parameters to change improved the fit; an 11% increase in GBB combined with a 0.4 mV hyperpolarization of the resting membrane potential (from a control group value of -82.6 to -83.0 mV, i.e., an increase in pump currents) resulted in a 77% overall reduction in discrepancy. The corresponding reductions in discrepancy for the SDTC, threshold electrotonus, current–threshold relationship, and recovery cycle, were 99.6, 87.3, 68.7, and 69.3%, respectively. The group mean responses for threshold electrotonus, current–threshold relationship, and recovery cycle are replotted in **Figures 4A–C** together with the best fit simulations. These modeling results indicate that an increase in leakage current under or through the myelin sheath is the most likely explanation for the fanning out of threshold electrotonus in the CP subjects. Combinations of changes in other parameters, together with an increase in GBB, did not improve the overall fit to the CP data (**Table 3B**). For instance, the reduction in GH to 5.15 from the control value of 5.5 (a possible explanation for the larger threshold increase in CP), resulted in a worse overall fit (72.7% reduction in discrepancy), especially hyperpolarizing threshold electrotonus (**Figure 5A**). The fits achieved by increasing nodal slow K^+^ conductance (GKsN) to 42.5 (**Figure 5B**), or decreasing the permeability of Na^+^ channels at the node (PNaN) to 4.05 (**Figure 5C**) did not improve the overall fit, especially subexcitability.

**Table 3:** Modeling axon excitability in CP and control groups.

(A) Best fits obtained by changing single parameters.

**Parameter**	**CP**	**Control**	**%**

GBB	38	35.8	61.9
GLkI	1.41	2.35	52.4
IPumpNI	0.0067	0	46.1
GH	4.6	5.5	41.0
GLkN	1.27	1.53	26.7
GKfI	120	145	6.4
**(B) Best fits obtained by changing two parameters.**

**Parameter 1**	**CP**	**Control**	**Parameter 2**	**CP**	**Control**	**%**

GBB	38.8	35.8	IPumpNI	0.0027	0	76.8
PNap(%)	0.83	0.86	GBB	40.3	35.8	76.5
PNaN	4.05	4.15	GBB	39.9	35.8	76.2
GKsN	42.5	40	GBB	40.2	35.8	75.8
GKfN	26.5	23.5	GBB	40.6	35.8	75.5
GH	5.15	5.5	GBB	38.4	35.8	72.7

*CP, Cerebral palsy; %, percentage reduction in discrepancy; GBB, Barrett–Barrett conductance across the myelin sheath; GLkI, internodal leak conductance; IPumpNI, nodal and internodal pump currents combined; GH, HCN channel conductance; GLkN, nodal leak conductance; GKfI, internodal fast K^+^ conductance; PNap(%), percentage of Na^+^ channels that are persistent; PNaN, permeability of Na^+^ channels at the node; GKsN, nodal slow K^+^conductance; GKfN, nodal fast K^+^ conductance.*

### Relationship between Nerve Excitability and Clinical Measures

**FIGURE 6:**
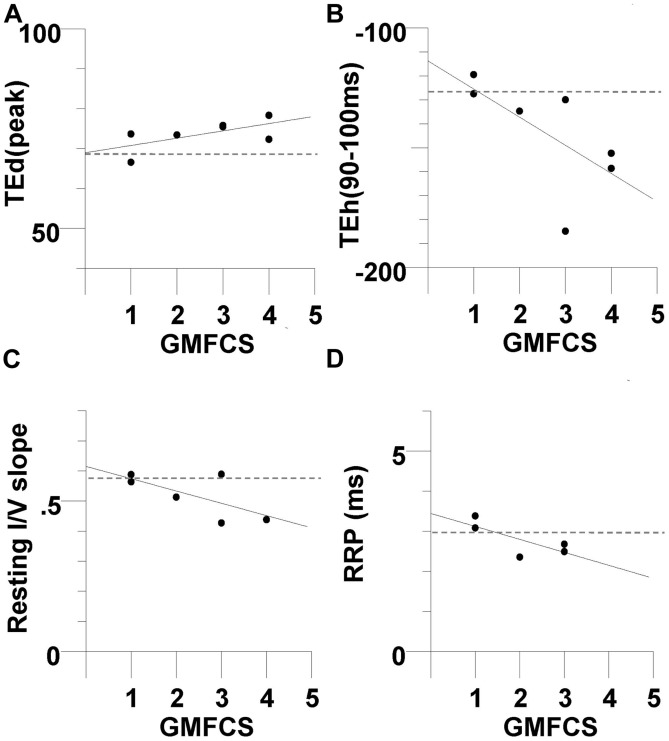
**Evidence of abnormal axon excitability in CP subjects with greater impairment in gross motor function**. There was a trend for excitability parameters to be related to Gross Motor Function Classification System (GMFCS) level, but the correlations were not statistically significant The CP subjects with a larger GMFCS level (greater motor impairment) tended to have larger +40% depolarizing electrotonus (*R* = 0.63, *P* = 0.12) **(A)**, larger -40% hyperpolarizing electrotonus (*R* = 0.65, *P* = 0.1) **(B)**, smaller resting I/V slope (*R* = -0.67, *P* = 0.13, *N* = 6) **(C)**, and smaller RRP (*R* = -0.74, *P* = 0.14, *N* = 5) **(D)**. The dashed lines are the control group means.

The relationship between nerve excitability parameters and clinical measures was determined to investigate whether axonal properties were related to motor impairment. Excitability parameters were found to be unrelated to the MACS, MAS, MRC, and MVC. However, there was a trend for excitability parameters to be related to GMFCS level, but the correlations were not statistically significant (**Figure 6**). The TEd(peak) and TEh(90–100 ms) were both associated with GMFCS, indicating that greater fanning out of threshold electrotonus occurred in subjects with poorer gross motor function (*R* = 0.63, *P* = 0.12, **Figure 6A** *R* = 0.65, *P* = 0.1, **Figure 6B**, respectively). Also, the resting I/V slope and the RRP were both smaller in those with poorer gross motor function (*R* = −0.67, *P* = 0.13, **Figure 6C**; *R* = −0.74, *P* = 0.14, **Figure 6D**, respectively). When compared to the mean values of the controls (dashed lines in **Figure 6**), abnormal axon excitability was evident in individuals with a GMFCS level III or IV.

## Discussion

On evidence that motoneurons are hyperexcitable in CP (Barolat-Romana and Davis, 1980; Futagi and Abe, 1985; O’Sullivan et al., 1998; Tekgul et al., 2013), we anticipated that axons would also be hyperexcitable. However, we found that CP axons were less excitable rather than more excitable compared to control; resting threshold and rheobase tended to be larger, and there was evidence of membrane hyperpolarization. The most conspicuous abnormality in the CP subjects was their larger threshold changes during depolarizing and hyperpolarizing electrotonus (**Figure 1C**). Given that threshold electrotonus mostly reflects internodal properties (Bostock and Baker, 1988), the findings indicate altered internodal behavior in CP.

The younger age of the CP than the control subjects is unlikely to explain their fanning out of threshold electrotonus. In previous studies, hyperpolarizing electrotonus was unrelated to age (Bostock et al., 1995; Jankelowitz et al., 2007b; Bae et al., 2008). This was also the case for depolarizing electrotonus in two reports (Bostock et al., 1995; Jankelowitz et al., 2007b). In another study, early depolarizing electrotonus (TEd10–30 ms) was also unrelated to age based on a regression analysis of subjects 20–86 years of age (Bae et al., 2008). However, TEd(90–100 ms) was smaller with increased age, although it is uncertain whether the means for the 30–39 year-old (48%) and 50–59 year-old (46%) groups (comparable to the age difference in the present study) differed significantly (Bae et al., 2008).

As listed in **Table 3B**, changes in ion conductance together with the increase in GBB may account for the excitability changes in CP, rather than the latter combined with membrane hyperpolarization. However, the 10% decrease (from 5.5 to 5.15) in the hyperpolarization-activated cation current (referred to as GH in the table) in CP compared to control is a less plausible explanation for fanning out because it should have resulted in a smaller hyperpolarizing I/V slope during strong currents (i.e., reflecting reduced inward rectification), but this was not the case (**Figure 2B**). Also, changing GH on its own resulted in a much poorer fit to the CP data than changing GBB alone (41% vs. 61.9% reduction in discrepancy, **Table 3A**). Similarly, the 10% increase in the nodal slow K^+^ conductance (GKsN) in CP is also less likely as it should have resulted in greater S2 accommodation and a larger depolarizing I/V slope (i.e., reflecting increased outward rectification), but neither of these changes was evident (**Table 2**). Finally, the 10% reductions in transient (PNaN) or persistent (PNap%) Na^+^ currents are also less likely as they should have resulted in a smaller superexcitability in CP compared to control, but the opposite was found (**Figure 3D**; **Table 2**; Kiernan et al., 2005). Together, the pattern of excitability responses and modeling results suggest a larger internodal leakage current and modest hyperpolarization of the resting membrane potential in the CP axons.

### Are Adult CP Motor Axons Immature?

The larger GBB in CP may reflect abnormal development of the internode as a result of the brain injury. During development Schwann cells communicate with the axon to help establish the myelin sheath (Michailov et al., 2004), and during this period myelination may be regulated by axonal impulse activity (Fields, 2004). Thus, it is possible that myelin and related structures did not fully develop (i.e., is immature) as a consequence of decentralization. Specifically, there may be a loosening of the axon-schwann cell paranodal seal or the myelin may be thinner, resulting in greater shunting of current under and through the myelin sheath (Rosenbluth et al., 2013). That the axons may be immature is supported by the observation that excitability properties of the CP subjects are similar to those in healthy children at an age when axons are not yet fully developed (Farrar et al., 2013). In that study, nerve excitability properties in subjects 0.5–25 years of age were determined. Younger age was found to be associated with less excitable and slower conducting axons, and the reported means for threshold electrotonus (i.e., TEh90–100 ms = -145.8%) and resting I/V slope (0.53) in the late childhood group (3–8 years) are similar to our CP mean values. In addition, their modeling analysis indicated larger GBB and fast K^+^ conductance in infants and children compared to the young adult group. Although there was no strong evidence of an increase in fast K^+^ conductance in our CP subjects, possibly indicating that the axon-schwann cell paranodal seal is more developed in adult CP than in healthy children, the overall pattern of the excitability properties is similar to the pattern recorded in late childhood (Farrar et al., 2013). Finally, patients with hereditary neuropathy with liability to pressure palsies, a disorder of myelination, also show similar changes in nerve excitability as the CP subjects (Farrar et al., 2014). These observations, together with evidence of slowed conduction velocity in children with CP (Lang et al., 1983), imply that abnormalities in myelination may arise early in CP and persist into adulthood.

### Motoneurons and Axons

Stretch reflex excitability has been found to decrease with development, i.e., from infancy to early childhood (O’Sullivan et al., 1998), and may be explained in part by a reduction in motoneuron excitability (Mellstrom and Skoglund, 1969; Kellerth et al., 1971). However, in many people with CP, the normal developmental reduction in reflex excitability does not occur, so that hyperexcitable reflexes emerge and persist into adulthood (Barolat-Romana and Davis, 1980; O’Sullivan et al., 1998; Achache et al., 2010). We anticipated that axons would be similarly more excitable in CP than control, but this was found not to be the case. Rather, the slight membrane hyperpolarization in the CP axons may represent an adaptation to offset heightened motoneuron excitability (Ng et al., 2008). We can only speculate that a similar hyperpolarization occurs in the axon initial segment (the site of action potential initiation), thus providing an axonal mechanism for long-term regulation of motoneuron output (Grubb et al., 2011). Interestingly, there are opposite changes in the excitability of motoneurons and axons during development. For example, between infancy and childhood, motoneurons become less excitable (i.e., reduced reflex excitability; O’Sullivan et al., 1998) at the same time that axons become more excitable (i.e., reduced rheobase, increased conduction velocity; Farrar et al., 2013). These opposite changes in excitability may indicate that axons influence (or are influenced by) cellular (i.e., motoneuron) and network (i.e., spinal) activity over the long term (Bucher and Goaillard, 2011).

### Clinical Implications

The subjects with the most abnormal excitability recordings tended to have the poorest gross motor function, although the correlations were not significant (**Figure 6**). Subjects 2, 3, and 6 demonstrated the largest fanning out (**Figures 6A,B**) and the smallest resting I/V slope (**Figure 6C**; the current–threshold relationship was not be recorded in subject 3). The GMFCS assesses self-initiated movements with an emphasis on function in sitting and walking. Persons rated as level I have the most independent motor function and those at level V have the least (Palisano et al., 1997). The apparent relationship between axon excitability and the GMFCS level may reflect the nature of the original injury as noted above; a more severe lesion resulting in more dependence and abnormal axon excitability. Alternatively, age-related deterioration of function and disuse may have caused axonal changes (Sandstrom et al., 2004; Peterson et al., 2012). In animal models, limb disuse results in thinner myelin and slower conduction velocity (Canu et al., 2009).

The increased shunting of current in the CP axons may leave them prone to activity(exercise)-induced membrane hyperpolarization (Park et al., 2011), and could be a factor in the persistent fatigue that occurs in this population (Jahnsen et al., 2003). The membrane potential is modulated in part by the electrogenic Na^+^-K^+^ pump, which expels three Na^+^ sodium ions from the cell for every two K^+^ ions brought in. The increase in axonal impulse traffic during exercise leads to Na^+^ accumulation. This Na^+^ accumulation increases pump activity resulting in membrane hyperpolarization and an increase in threshold current (i.e., the threshold may increase by 40% during a 1 min maximal contraction; Park et al., 2011). Conceivably, the larger nodal (Na^+^) currents required to activate CP axons (i.e., because of current shunting via the GBB) would increase pump activity and lead to greater activity-induced membrane hyperpolarization compared to control axons. Given that the safety margin for conduction at each node of Ranvier is normally high (i.e., five times more current is generated than required to produce an action potential), the shunting of current may not necessarily lead to conduction block (Park et al., 2011). However, rehabilitative modalities that depend on stimulation of peripheral nerves such as functional electrical stimulation may be directly affected by current shunting and other changes in axonal properties (Wright and Granat, 2000). Further work is needed to test activity-induced changes in axonal properties and their association with fatigue in CP (Jahnsen et al., 2003).

The application of nerve excitability testing as part of the functional assessment and rehabilitation of patients with neurological disorders is in its infancy. The assessment of axonal function may have a role in monitoring the effectiveness of drugs on weakness and spasticity, common impairments in CP (Kuwabara and Misawa, 2004). Given, the recent evidence of motor unit loss in adult CP (Marciniak et al., 2015), new drugs that minimize axonal loss and/or changes in myelin may be a fruitful area for investigation. The effects of rehabilitation or exercise training on axonal properties *in vivo* are largely unexplored. However, a recent study found that some of the excitability changes associated with spinal cord injury could be reversed with 6 weeks of peripheral nerve stimulation (5 days per week of intermittent 100 Hz stimulation for 30 min; Lee et al., 2015). Conceivably, a similar type protocol or voluntary exercise may ameliorate the changes in axonal properties in CP. Given the sensitivity of myelin to changes in limb use, exercise may prove effective at increasing myelin thickness (Canu et al., 2009).

### Limitations

Given the small number of CP subjects studied, the findings may not necessarily reflect axonal properties in the wider CP population. Most of the CP subjects studied had quadriplegia. It is uncertain whether similar changes in axon excitability would occur in other patterns of injury (i.e., diplegic CP, the affected limb in hemiplegic CP). As this was a cross-sectional study of adults, we cannot determine conclusively whether the axonal changes in CP arose early in life around the time of their injury or developed in adulthood. Clearly, further study of CP is necessary to establish the time course of axonal changes and how they relate to the evolution of motor impairment. A longitudinal maturational study of axon excitability in CP is feasible given the recent study in healthy infants and children (Farrar et al., 2013).

## Conclusion

The results indicate abnormal internodal properties in CP that may be attributed to altered architecture. The findings are consistent with other studies of central motor disorders in that axon excitability far removed from the lesion is altered, and may reflect a lasting signature of decentralization.

## Conflict of Interest Statement

The authors declare that the research was conducted in the absence of any commercial or financial relationships that could be construed as a potential conflict of interest.
